# Effects of Iranian Pomegranate Peel Anthocyanin Extract on Physicochemical, Microbial, Sensory, and Shelf Life Properties of Set Yogurt

**DOI:** 10.1002/fsn3.70440

**Published:** 2025-06-20

**Authors:** Niloofar Zahed, Reza Esmaeilzadeh Kenari

**Affiliations:** ^1^ Department of Food Science and Technology Sari Agricultural Sciences and Natural Resources University Sari Iran

**Keywords:** antimicrobial activity, antioxidant activity, fermented product, microwave‐assisted extraction, natural pigment, pomegranate peel

## Abstract

The study aimed to add the optimal concentration of Iranian pomegranate peel anthocyanin extract (PoPAE) to the formulation of set yogurt. Then, the physicochemical, microbial, and sensory characteristics of set yogurt containing pomegranate peel anthocyanin extract (YCPAE) were compared with set yogurt without extract (YWPAE) during the storage period (12 days). The amount of anthocyanin and total phenol measured equaled 73.57 mg cyanidin‐3‐glucoside/100 g extract and 2153.11 mg gallic acid/100 g extract. The concentrations of 600 ppm (95.83%) and 800 ppm (96.57%) of PoPAE showed the highest DPPH radical inhibitory power (*p* < 0.05). During storage days (1, 4, 8, 12), YCPAE parameters such as pH, syneresis, antioxidant activity (DPPH, FRAP, and β‐carotene: linoleic acid (βC/LA) bleaching), and antimicrobial activity (mold, yeast, *Staphylococcus aureus* , and *Pseudomonas aeruginosa* ) and a* index were higher than those of YWPAE. However, parameters such as acidity, viscosity, index *L*, and b* of YCPAE were lower than those of YWPAE in days of storage (1, 4, 8, 12). The smell and taste in YCPAE on the 12th day scored high. Scores for color, texture, and mouth feel decreased over time. The texture, smell, taste, and mouth feel of YWPAE on the 12th day scored more points than YCPAE. Looking ahead, the findings strongly suggest that YCPAE will transform its market presence by not only extending the product's shelf life but also significantly enhancing key physicochemical properties. Its remarkable antioxidant properties, combined with its essential nutritional benefits, promise to have a significant impact on consumer health.

## Graphical abstract

During storage days (1, 4, 8, 12), YCPAE parameters such as pH, syneresis, antioxidant activity (DPPH, FRAP, and β‐carotene: linoleic acid bleaching), and antimicrobial activity (mold, yeast, *Staphylococcus aureus* , and *Pseudomonas aeruginosa* ), a* index had a higher value than the YWPAE. However, parameters such as acidity, viscosity, index *L*, and b* of YCPAE were lower than those of YWPAE. The smell and taste in YCPAE on the 12th day scored high.

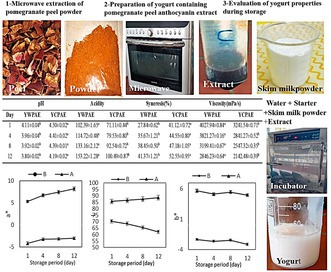

## 1 Introduction

Yogurt is considered a fermented product and is one of the most popular dairy products worldwide. Yogurt is in high demand due to its beneficial compounds, various vitamins and minerals, and positive health effects (Weerathilake et al. 2014). Yogurt improves lactose tolerance, strengthens the immune system, and prevents digestive problems (Ndife et al. 2014). Awareness of the valuable properties of yogurt has led to growing consumer demand (Weerathilake et al. 2014).

The most important among the various additives in food products is plant pigment with antioxidant properties. In addition to being visually appealing and a new taste for the consumer, it is also healthier than synthetic pigments (Gawai et al. 2017).

Consumer interest in fermented dairy products, particularly yogurt, has increased. The use of synthetic compounds (synthetic dyes) in yogurt has raised concerns due to adverse effects, including hyperactivity in children and the risk of cancer. It is necessary to use natural colors and flavorings (food waste) that have high antioxidant and antimicrobial properties, which, in addition to having no harmful effects, because of their antioxidant properties, prevent many diseases, including heart disease, cancer, and diabetes, and are useful against diseases. (Golmakani et al. 2021).

Pomegranate ( *Punica granatum* L.) is cultivated in different continents, including Asia, Europe, America and Africa (Farahmand et al. 2017). Iran ranks first in pomegranate production in the world, followed by countries such as the United States, Turkey, Egypt, Italy, and India (Troujeni et al. 2018; Farahmand et al. 2017). Catechins, flavonoids, and cyanidins are found in abundance in the peel, fruit, and seeds of pomegranate. These compounds offer practical benefits, serving as natural food additives, preservatives, and enhancing the nutritional profile and shelf life of various food products. Additionally, they have medicinal properties, including antioxidant, anti‐inflammatory, antimicrobial, anticancer, and cardioprotective effects (Golmakani et al. 2021; Siddiqui et al. 2024). Plant extracts have high antioxidant properties because in low concentrations, they proved the ability to compete with synthetic antioxidants such as TBHQ.

Fresh fruit juice is the most common product prepared from pomegranate fruit. Pomegranate peel and core are considered waste. The peel of this fruit has the highest ranking compared to the peel and seeds of other plants due to its unique antioxidant power (Kaderides et al. 2015). In various food products, including jams and juices, sauces, sweets and candies, dairy products (yogurt and milk), and frozen products (ice cream), plant anthocyanins are used as natural food pigments (Sahat et al. 2014).

Researchers have reported mixed results in studies on the properties of yogurt containing pomegranate peel anthocyanin extract. They stated that pomegranate peel extract maintains starter bacterial strains and probiotic bacteria, but results in an undesirable taste (Jany et al. 2024), texture, and appearance (Ibrahim et al. 2020). However, it is effective in eliminating harmful bacteria and fungi in yogurt (Alsubhi et al. 2022). On the other hand, some researchers have reported that the addition of pomegranate peel extract is associated with a decrease in antioxidant activity (Kharchoufi et al. 2017), a sharp decrease in pH, and high synergism during product storage (Bakhti et al. 2025). According to studies conducted on yogurt containing pomegranate peel extract, the challenge of precise formulation to produce a high performance product while maintaining appropriate organoleptic, physicochemical, and marketability properties remains. Therefore, the extraction of bioactive compounds from pomegranate peel requires careful selection of techniques to maximize yield and quality. Green extraction methods, including pressurized liquid extraction, ultrasound‐assisted extraction, microwave‐assisted extraction, and enzyme‐assisted extraction, offer efficient and sustainable alternatives to traditional methods (Singh et al. 2023). Based on our previous research on the extraction of pomegranate peel extract by different methods, such as ethanol solvent, ethanol‐water solvent, ultrasound, and microwave, the results showed the superiority of the extract obtained by microwave extraction in terms of phenolic compounds, anthocyanins, and antioxidant properties (Zahed et al. 2023). Researchers confirmed the superiority of ethanol solvent over water for the extraction of phenolic compounds from pomegranate peel. However, the nature of the phenolic compounds present in the extract and their antioxidant activity remain the determining elements for the choice of solvent (Kennas and Amellal‐Chibane 2019). Motikar et al. (2021) reported the superiority of microwave over ultrasound for the extraction of phenolic compounds with high antioxidant properties. Researchers also showed that the use of ethanol is more efficient than water in green extraction methods (ultrasound) for the preparation of pomegranate peel extract (Pore et al. 2024). Although water is more polar than ethanol, ethanol is superior to water in separating phenolic compounds and anthocyanins due to its organic nature (Masci et al. 2016).

On the other hand, no study has been reported so far on the addition of pomegranate peel anthocyanin extract obtained by microwave extraction (with ethanol solvent) to a yogurt set. Therefore, in this study, anthocyanin extract was extracted from pomegranate peel powder using microwave (with ethanol solvent). The amounts of anthocyanin, phenolic compounds, and antioxidant properties were changed. Then, the optimal concentration of the extract was added to the yogurt set formulation. In this study, the physicochemical properties, antioxidant properties, antimicrobial activity, and sensory properties of pomegranate peel anthocyanin extract yogurt and control yogurt were evaluated and investigated from the time of production and during storage (12 days).

## 2 Materials and Methods

### 2.1 Materials

Pomegranate fruit was purchased from the orchards of Kordkuy city (Iran). Hansen's starter was purchased from Denmark, and skimmed milk was purchased from Parsi Powder Store (Hamadan, Iran). All chemical and nonchemical materials were obtained from Merck and Sigma‐Aldrich.

### 2.2 Methods

#### 2.2.1 Pomegranate Peel Powder

The peel was separated from the pomegranate fruit (washed with distilled water). The peel dried in the shade at 27°C for 72 h. A mill crushed it, and the pomegranate peel powder was separated by a sieve (mesh 40) and kept in the refrigerator (4°C) for use.

#### 2.2.2 Microwave Extraction

The method chosen for this experiment was based on the results of our previous research, which was a comparison between classical and green extraction in terms of extraction conditions and solvent‐to‐sample ratio. The best extraction in terms of efficiency and amount of phenolic and antioxidant compounds was related to microwave extraction. The obtained extract had the highest values of TAC and TPC. On the other hand, the presence of acidic compounds harms yogurt starter bacteria. As a result, acidic and nonacidic ethanol extraction were compared in terms of effective compounds, and microwave extraction with ethanol solvent (without using acidic compounds) was the superior sample and was therefore used in this study. Pomegranate peel powders (5 g) were mixed with 96% ethanol (100 mL), and then the mixture of peel and ethanol was placed in the microwave device for 5 min. The microwave power and temperature were set to 360 W and 45°C, respectively. The extract was centrifuged by a refrigerated centrifuge (5°C:8 min:8000 *g*). Next, the upper phase was transferred into the beaker, which was then placed in an oven set to 45°C. Until the ethanol evaporates. Then, PoPAE was kept in a freezer (−20°C) (Zahed et al. 2023).

##### 2.2.2.1 Total Anthocyanin Content (TAC)

The TAC of PoPAE was determined using the differential pH method. Some distilled water (99 mL) was mixed with PoPAE (1 mL). Then, the buffer solution of KCl and C2H3NaO2 was mixed with PoPAE with a ratio of 1:4. The prepared sample was kept in it for 20 min. The wavelength of the spectrophotometer was set to 520 and 700 nm. The final results were determined from the equation written below (Kırca and Cemeroğlu 2003).

$$A=\left(A_{52\text{0nm}}-A_{70\text{0nm}}\right)\;{\mathrm{pH}}_{1.0}–\left(A_{52\text{0nm}}–A_{70\text{0nm}}\right)\;{\mathrm{pH}}_{4.5}$$

$$\mathrm{TAC}=\left(A\times \mathrm{MW}\times \mathrm{DF}\times 10^{3}\times V\right)/\left(ɛ\times L\times M\right)$$

##### 2.2.2.2 Total Phenol Content (TPC)

First, the required solutions were prepared (the folin phenol reagent diluted 10 times (A) − 7.5% Na2CO3 solution (B)). Next, solution A and solution B in a ratio of 1:4 and 2000 ppm PoPAE were mixed. The final composition containing anthocyanin extract was kept in a greenhouse (25°C:30 min). Absorption was determined by a spectrophotometer (λ = 760 nm). The final results were determined from the equation written below (Singh et al. 2002).

$$Y=X0.8085X+0.0418\;R^{2}=0.990$$

*Y* = absorption, X = sample concentration, *R* = correlation coefficient.

##### 2.2.2.3 Inhibition of DPPH Assays

In this test, 100 μL of PoPAE with different concentrations (100, 200, 400, 600, and 800 ppm) was mixed with 10 mL of methanol (Contains 0.004% DPPH). After incubation (30 min:25°C), the optical absorption (λ = 517 nm) of anthocyanin extract samples of pomegranate peel powder was read against the control sample (0.004% DPPH solution in methanol). The control sample in this test was TBHQ (ppm 100). The inhibition percentage of DPPH free radical by PoPAE was declared using the following equation (Saviz et al. 2015):

$$\text{Inhibition}\%=\left(\left(\text{Ablank}-\text{Asample}\right)/\text{Ablank}\right)\times 100$$

To carry out the next stages of the research, we performed a DPPH free radical inhibition test using different concentrations of anthocyanin extract. We chose the lowest concentration that exhibited the most inhibition and antioxidant properties as the optimal concentration to add to set yogurt.

#### 2.2.3 Preparation of Set Yogurt Containing Anthocyanin Extract

First, skim milk powder was mixed with distilled water (dry matter 10%). It was kept in the refrigerator for hydration (1 h). Then, it was pasteurized for 5 min at 90°C. The anthocyanin extract (600 ppm) was added to the milk after lowering the temperature (40°C). Next, yogurt starter (3%) was added to the milk containing the extract (40°C) (100 mL). Next, it was placed in an incubator (3–5 h:40°C) to form a yogurt gel network. The formed yogurt was kept at refrigerator temperature.

##### 2.2.3.1 pH Determination

The pH of YCPAE and YWPAE was measured with a pH meter (SARTORIUS, Germany) (Karaaslan et al. 2011).

##### 2.2.3.2 Color Attributes

Indices such as *L*, a*, b* for YWPAE and YCPAE samples were evaluated by HunterLab colorimeter (Color Quest XE, USA) (Karaaslan et al. 2011).

##### 2.2.3.3 Acidity

The total titratable acidity of YWPAE and YCPAE was measured according to the Golmakani et al. (2021) method.

##### 2.2.3.4 Syneresis

Forty grams of the sample was poured into a special centrifuge container, and the separation process (1971 g) was carried out at a temperature of 10°C and a duration of 3 min. The weight of the clear upper phase was measured. The percentage of syneresis was obtained by dividing the weight of the upper phase by the initial weight of the yogurt (Wang et al. 2020).

##### 2.2.3.5 Viscosity

The viscosity of YWPAE and YCPAE during the storage period (12 days) was measured using a Brookfield viscometer at room temperature.

##### 2.2.3.6 Inhibition of DPPH Assays

YCPAE and YWPAE (100 μL) were mixed with 10 mL methanol (Contains 0.004% DPPH). The prepared samples were kept in it for half an hour. The wavelength of the spectrophotometer was set to 517 nm. The % inhibition was obtained according to the following equation (Saviz et al. 2015):

$$\text{Inhibition}\%=\left(\left(\text{Ablank}-\text{Asample}\right)/\text{Ablank}\right)\times 100$$

##### 2.2.3.7 βC/LA Bleaching Assay

The process of bleaching samples YCPAE and YWPAE was determined using the method developed by Razavi and Kenari (2021). First, the final solution was prepared, consisting of a mixture of beta‐carotene, chloroform, linoleic acid, and Tween 40. The aforementioned compounds were poured into a special test container in amounts of 1 mg, 2 mL, 50 μL, and 400 μL, respectively, and mixed. The prepared solution was shaken for further mixing. The next step was to add 720 μL of the final solution prepared in a special test container. 80 μL of YCPAE and YWPAE at a concentration of 600 ppm were combined with the final solution in a special test container. The water bath was set at 50°C. The prepared samples were kept in it for 2 h. The wavelength of the spectrophotometer was set to 470 nm. The antioxidant capacity of the samples under study was determined as the percentage of inhibition using the following equation.

$$\text{Inhibition}\%=\left(\left(\mathrm{Ac}-\mathrm{Ad}\right)/\mathrm{Ac}\right)\times 100$$

##### 2.2.3.8 Ferric Reducing Antioxidant Power (FRAP) Assay

The method of Agregán et al. (2017) was used to measure FRAP. For this purpose, the solutions required for the experiment (300 mM acetate buffer solution (pH = 3.6): 40 mM hydrochloric acid (containing 1 mM TPTZ): 20 mM FeCl3·6H2O) were prepared and combined in a ratio of 10:1:1 (v:v:v), respectively. Next, YCPAE and YWPAE at a concentration of 600 ppm were combined with 300 μL of FRAP solution and placed in an incubator at 37°C. The wavelength of the spectrophotometer was set to 593 nm. Eight minutes after the preparation of the desired sample, the absorbance was determined. The antioxidant power of YCPAE and YWPAE was mentioned in terms of Ferric (μmol)/mg.

##### 2.2.3.9 Microbiological Analysis

The culture media related to mold, yeast, *Staphylococcus aureus* , and *Pseudomonas aeruginosa* were prepared according to the manufacturer's instructions for the culture medium. Dilution (10^−6^) of YCPAE and YWPAE was done. The yeast and mold culture medium was incubated in potato dextrose agar (aerobic conditions) for 3 days at 32°C. The two strains were counted separately (Wijesekara et al. 2022). *S. aureus* and *P. aeruginosa* were incubated in MSA and PAB medium and kept at 37°C for 24 h. The colony counts of molds, yeasts, *S. aureus* , and *P. aeruginosa* were counted in 10 log (CFU/mL) colony forming units (Motawee and Neveen 2016).

##### 2.2.3.10 Sensory Characteristic

The sensory characteristic form was based on Golmakani et al. (2021). Sensory evaluation was done by 10 trained panelists (five women and five men in the age range of 20–30 years). Five marks were used for evaluation. A score of 0 indicates the most unfavorable, and a score of five indicates the most favorable result. Panelists drank water between samples to reduce sensory evaluation error. The temperature and relative humidity of the test environment were 27°C and 66%, respectively. The panelists scored the mouthfeel, color, smell, texture, and taste of YCPAE and YWPAE samples.

In all tests, YCPAE and YWPAE were kept at refrigerator temperature for 12 days. To perform the tests, sampling was done on days 1, 4, 8, and 12 from YCPAE and YWPAE.

### 2.3 Statistical Analysis

One‐way analysis of variance (ANOVA) was used to analyze the data obtained in this study. The analysis was done to demonstrate the minimum significant difference at a confidence level of more than 95% (*p* < 0.05). The Duncan test was used for this purpose. The final results were presented as means ± SD. All tests except sensory properties were performed in triplicate. The graph was created using Excel software version 2016. In this study, SPSS 26 software was used.

## 3 Results and Discussion

### 3.1 Characteristics of PoPAE

#### 3.1.1 TAC and TPC

The results of this test (Table 1) proved that the TPC (1973.02 mg GA/100 g E) of PoPAE is more than its TAC (57.59 mg of C3G/100 g E) and had a significant difference (*p* < 0.05). Ethanol solvent combined with microwave application has higher performance in extracting phenolic compounds and anthocyanins because factors such as polarity, ionicity of bioactive substances, and wall permeability were affected by microwave. Azarpazhooh et al. (2019) stated TAC in the pomegranate peel extract to be 40.2 mg of C3G. Also, in other research, researchers stated that the extract of pomegranate peel with ethanol solvent has more phenolic compounds than the ethanol‐water mixture (Zahed et al. 2023). Ahmed and Abd Elhafez (2025) reported the amount of phenol obtained from pomegranate peel as 224.42 mg GA/100 mg E. On the other hand, researchers reported the amount of phenol obtained from pomegranate peel with methanol and ethanol solvents as 603 and 579.9 mg GA/100 g E, respectively (Lia and Tang 2024). The results of our study emphasized the importance of extraction with a green method, such as microwave, which can separate more phenolic compounds. On the other hand, the extract obtained from microwave can have higher antioxidant and antimicrobial activity than other extractions.

**TABLE 1:** Total phenol content and total anthocyanin content of pomegranate peel anthocyanin extract.

TPC (mgGAE/100 g E)	TAC (mgC3G/100g E)
2153.11 ± 12.03^a^	73.57 ± 2.41^b^

*Note:* Different letters in the row indicate significant differences (*p* < 0.05, *n* = 3).

Abbreviations: C3G, cyanidin‐3 glucoside; E, extract; GAE, gallic acid equivalent; TAC, total anthocyanin content; TPC, total phenol content.

#### 3.1.2 DPPH Assay

**FIGURE 1:**
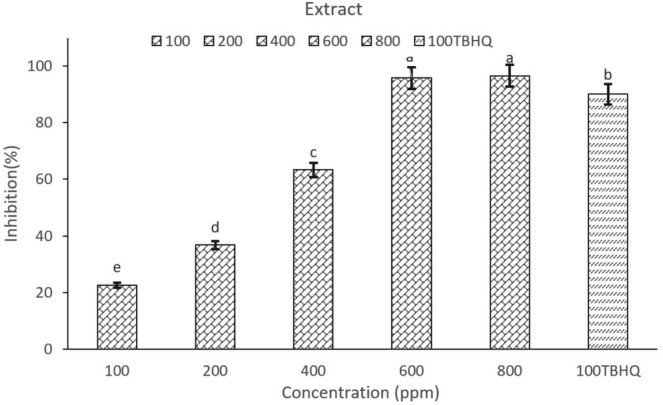
DPPH free radical inhibition percentage by pomegranate peel extract in different concentrations and synthetic antioxidant TBHQ. 100 (Anthocyanin extract of pomegranate peel with a concentration of 100 ppm), 200 (anthocyanin extract of pomegranate peel with a concentration of 200 ppm), 400 (anthocyanin extract of pomegranate peel with a concentration of 400 ppm), 600 (anthocyanin extract of pomegranate peel with a concentration of 600 ppm), 800 (anthocyanin extract of pomegranate peel with a concentration of 800 ppm), 100TBHQ (antioxidant TBHQ with a concentration of 100 ppm). Different letters indicate significant differences (*p* < 0.05, *n* = 3).

Pomegranate peel anthocyanin extract showed the highest inhibitory effect at concentrations of 600 ppm (95.83%) and 800 ppm (96.57%) (Figure 1), which were similar to each other (*p* < 0.05). Antioxidant TBHQ, with a concentration of 100 ppm (90.09%) had a lower inhibitory percentage compared to concentrations of 600 ppm and 800 ppm. The IC_50_ level of anthocyanin extract was equal to 302.89 ± 0.21 ppm, which indicates the high inhibitory power of the extract. Alsubhi et al. (2022) reported an IC50 of 200 ppm for pomegranate peel extract. It illustrates how extraction conditions, such as the type of solvent and extraction duration, affect the antioxidant compounds in the extract and their ability to inhibit free radicals because the extraction conditions have a direct effect on the type and amount of bioactive compounds, such as phenols, flavonoids, and anthocyanins. The majority of the antioxidant properties of pomegranate peel extract are related to cyanidin 3‐glucoside (anthocyanin) and gallic acid (phenolic compound) (Kharchoufi et al. 2017). Additionally, the results indicated that as the concentration of the extract increased, the inhibitory percentage of the extract also rose.

### 3.2 Choosing the Optimal Concentration of PoPAE

Based on the results obtained from the DPPH antioxidant test, the optimal concentration was used in the set yogurt formulation. The highest inhibition percentage of PoPAE in this test was with concentrations of 600 ppm and 800 ppm. No significant difference was observed between them (*p* < 0.05). Therefore, the concentration of 600 ppm of PoPAE was chosen as the optimal concentration to be added to the set yogurt formulation.

### 3.3 Set Yogurt Characteristics Analysis

#### 3.3.1 pH

The results indicated that the pH of YCPAE was consistently higher than that of YWPAE over the storage days (1, 4, 8, and 12) (Table 2). Also, with the increase in storage time, the pH of YCPAE and YWPAE decreased. The highest and lowest pH values were related to YCPAE on the first day (4.50) and YWPAE on the 12th day (3.80). In a study similar to the current study, the initial pH values of freshly prepared yogurt containing pomegranate peel extract and control were 4.48–4.64 on the first day. On the last day of storage (21), the pH was 4.15 and 3.4, respectively (Bakhti et al. 2025). In a study on the impact of varying concentrations of pomegranate juice in both set and stirred yogurts, it was observed that the addition of pomegranate juice significantly influenced the pH level of the yogurt. In contrast, adding pomegranate juice to yogurt had negative effects on the number of starter cultures due to damage caused by acids (Golmakani et al. 2021), aligning with the current study's findings. Because YCPAE was kept in an incubator for a longer time to reach the standard pH. Szołtysik et al. (2021) conducted an analysis of yogurt enriched with a dry polyphenolic extract from grape berries and starch. Their results showed that the pH level of the control yogurt sample was lower than that of the yogurt sample containing the extract, both initially and during storage. Following the findings of the current study, a greater decrease was observed at 14 days.

**TABLE 2:** pH, acidity, syneresis, and viscosity of YCPAE and YWPAE during storage (12 days).

Day	pH		Acidity		Syneresis (%)		Viscosity (mPa/s)	
	YWPAE	YCPAE	YWPAE	YCPAE	YWPAE	YCPAE	YWPAE	YCPAE
1	4.11 ± 0.04^b^	4.50 ± 0.02^a^	102.39 ± 1.65^a^	71.11 ± 0.44^b^	27.84 ± 0.62^b^	41.12. ± 0.72^a^	4027.94 ± 0.84^a^	3241.54 ± 0.71^b^
4	3.96 ± 0.04^b^	4.41 ± 0.02^a^	114.72 ± 0.48^a^	79.53 ± 0.80^b^	35.67 ± 1.21^b^	44.55 ± 0.80^a^	3821.27 ± 0.16^a^	2841.27 ± 0.52^b^
8	3.92 ± 0.02^b^	4.39 ± 0.01^a^	133.16 ± 2.12^a^	92.54 ± 0.72^b^	38.45 ± 0.50^b^	47.18 ± 1.05^a^	3199.41 ± 0.67^a^	2547.32 ± 0.35^b^
12	3.80 ± 0.02^b^	4.19 ± 0.02^a^	153.22 ± 1.28^a^	100.49 ± 0.87^b^	41.37 ± 1.21^b^	52.55 ± 0.95^a^	2846.23 ± 0.64^a^	2142.48 ± 0.39^b^

*Note:* Different letters in the row indicate significant differences (*p* < 0.05, *n* = 3).

Abbreviations: YCPAE, yogurt containing pomegranate peel anthocyanin extract; YWPAE, yogurt without pomegranate peel anthocyanin extract.

#### 3.3.2 Acidity

The results indicated that the acidity of YCPAE was consistently higher than that of YWPAE over the storage days (1, 4, 8, and 12) (Table 2). Also, the acidity of YCPAE and YWPAE increased with the increase in storage days. The lowest and highest amounts of acidity were related to YCPAE on the first day (71/11) and YWPAE on the 12th day (153/22). Szołtysik et al. (2021) found that yogurt enriched with dried grape berry polyphenolic extract did not show significant changes in physicochemical properties, for example, acidity and rheological parameters. Machado et al. (2023) stated that yogurt containing anthocyanin extract of *jabuticaba* peel was similar in acidity and pH to the commercial sample. Ahmed and Abd Elhafez (2025) reported that the acidity of yogurt containing 1.5% pomegranate peel (112) was lower than that of control yogurt (161). However, they reported a significant increase in acidity during storage. On the 21st day of storage, they reported the acidity of yogurt containing pomegranate peel and control as 212 and 192, respectively. The high amount of pomegranate peel extract in the yogurt formulation led to the acidity of yogurt, which resulted in consumer dissatisfaction.

#### 3.3.3 Syneresis

The results (Table 2) showed that syneresis increased with the passage of storage time of YCPAE and YWPAE. The lowest and highest amount of syneresis was related to YWPAE on the first day (27.84%) and YCPAE on the 12th day (52.55%). The results showed that the syneresis of YCPAE was higher than that of the control sample in storage days (1, 4, 8, and 12). Golmakani et al. (2021) stated that high concentrations of pomegranate juice in yogurt had a direct relationship with increased acidity and decreased pH. Finally, it increased the syneresis of yogurt, aligning with the current study's findings. Jany et al. (2024) reported the syneresis of control yogurt and yogurt mixed with pomegranate peel extract as 68% and 59%, respectively, which is higher than the syneresis of our yogurt. Factors such as the percentage of dry matter (protein), the strain of starter bacteria, and the acidity of yogurt play a key role in reducing syneresis.

#### 3.3.4 Viscosity

The viscosity of YCPAE was lower than that of YWPAE during storage (Table 2), and significant differences were observed. As storage time increases, the viscosity of both YCPAE and YWPAE decreases. The highest and lowest viscosities were related to YWPAE on the first day (4027.94) and YCPAE on the 12th day (2142/48). Researchers reported that increasing the concentration of pomegranate extract in yogurt resulted in a decrease in viscosity (El‐Said et al. 2014). Machado et al. (2023) observed that adding jabuticaba peel extract to yogurt resulted in a decrease in viscosity. A decline in viscosity was observed in yogurt that contained dried grape berry polyphenolic extract. During a storage period of 14 days, both the yogurt with the extract and the control yogurt showed a reduction in viscosity (Szołtysik et al. 2021), which aligns with the findings of the current study. Temiz and Ersöz (2023) stated that adding 300 mg/L of pomegranate extract to yogurt augmented syneresis and reduced product viscosity. However, it was within the standard range. If pomegranate peel extract is added to yogurt at a higher concentration (1%), it will lead to significant changes in the syneresis and viscosity parameters of yogurt, because the interaction between casein and phenolic compounds is an important factor in the stability of the gel network structure of yogurt (Yağmur et al. 2023). Ibrahim et al. (2020) stated that the viscosity of probiotic yogurt containing pomegranate peel increased during storage. The presence of pectin in pomegranate peel led to an increase in the viscosity of probiotic yogurt.

#### 3.3.5 Color Attributes

**FIGURE 2:**
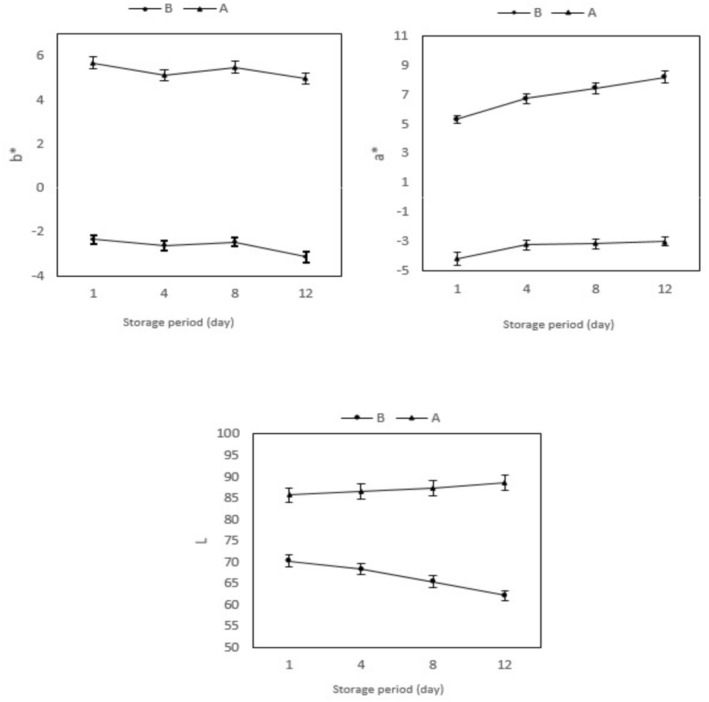
*L*, a* and b* index for measuring color YCPAE (B) and YWPAE (A) during the storage period (12 days). YCPAE (B) (ogurt containing pomegranate peel anthocyanin extract), and YWPAE (A) (yogurt without pomegranate peel anthocyanin extract). Different letters indicate significant differences (*p* < 0.05, *n* = 3).

YWPAE has a higher L parameter during storage than YCPAE (Figure 2). On the other hand, the whiteness of YCPAE decreased during storage. YWPAE had a negative value for parameter a*. It was due to the presence of vitamin B12 (pale green color) in yogurt. The anthocyanin present in the extract has led to the positive a* parameter in YCPAE. On the other hand, YCPAE increased its redness during storage. As time progressed, the pH of the yogurt gradually lowered from 4.5, leading to an enhancement in the color of anthocyanin and an improvement in its stability. This shift not only intensified the visual appeal of the yogurt but also contributed to the overall quality and longevity of its anthocyanin content. YWPAE had a positive value for the b* parameter. But YCPAE had a negative value for the b* parameter. Because the anthocyanin present in the pomegranate peel includes various anthocyanidins, which have hydroxyl groups in their structure, leading to a slight blue color in the sample. By decreasing pH and increasing acidity during storage, the amount of blue color of YCPAE increased during storage. Blueberry extract is a source of natural colors and composition in yogurt. The quality indices of color, such as *L*, a*, and b* did not have significant differences after 7 days. The pH was constant for 7 days (Pires et al. 2020), but in the current research, the pH decreased with the passage of storage time. The change had a beneficial impact on the stability of the anthocyanin pigment. Sahingil and Hayaloglu (2022), enriching fortified yogurt (5%–20%) with rose flowers, observed that color quality indices such as *L* and b* decreased, and a* remained constant. In research by Machado et al. (2023) on yogurt containing jabuticaba peel powder extract, it was stated that the extract was the factor that reduced the a* parameter of yogurt. Researchers reported that PoPAE in strawberry yogurt smoothie resulted in maintaining the red color during storage compared to the control sample (Alsubhi et al. 2022). It can be concluded that the changes in parameters (*L*, a*, b*) are affected by the type of extract, the amount of extract, the percentage of dry matter, fat and water content, and the pH of the yogurt during the storage period.

#### 3.3.6 DPPH Assay

**FIGURE 3:**
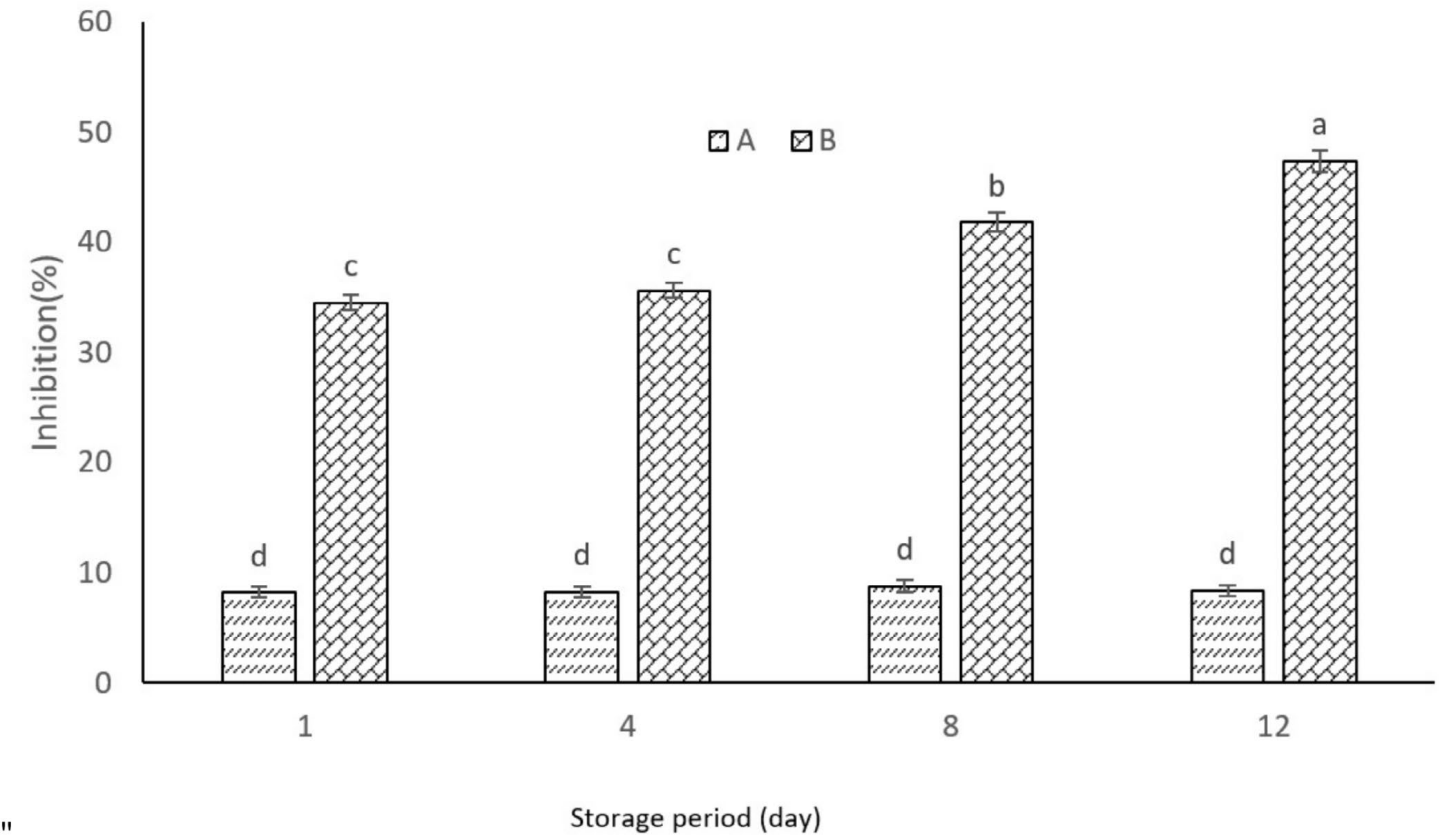
DPPH free radical inhibition percentage in YCPAE (B) and YCPAE (A) during the storage period (12 days). YCPAE (yogurt containing pomegranate peel anthocyanin extract) and YWPAE (yogurt without pomegranate peel anthocyanin extract). Different letters indicate significant differences (*p* < 0.05, *n* = 3).

YCPAE showed high antioxidant properties and had a significant difference with YWPAE (Figure 3). Because the anthocyanin in YCPAE has high antioxidant properties. Also, the results show that the antioxidant property of the sample increased during storage. However, similar results were observed on days 1 and 4 (*p* < 0.05). With storage time, changes in acidity and pH resulted in more active and stable anthocyanins, exhibiting greater antioxidant properties. Researchers noted that yogurt containing pomegranate peel extract in varying proportions (5%–35%) had unique inhibitory and antioxidant power compared to control yogurt. It was also directly related to increasing the extract ratio in yogurt (El‐Said et al. 2014). Similar to the current study, Sahingil and Hayaloglu (2022) confirmed that yogurt containing the extract had higher antioxidant power with increasing storage days (15 days). Researcher findings revealed that the addition of pomegranate juice significantly enhances antioxidant activity, making it not only a flavorful addition but also a highly nutritious one (Golmakani et al. 2021). This study highlights pomegranate juice as an exceptional option for creating flavored yogurts that boast impressive health benefits. Phenolic compounds have greater antioxidant activity in acidic solution (El‐Said et al. 2014) because pH affects the ionization state of bioactive phenolic compounds (Kharchoufi et al. 2017). This explains the increase in antioxidant activity during storage.

#### 3.3.7 βC/LA Bleaching Assay

**FIGURE 4:**
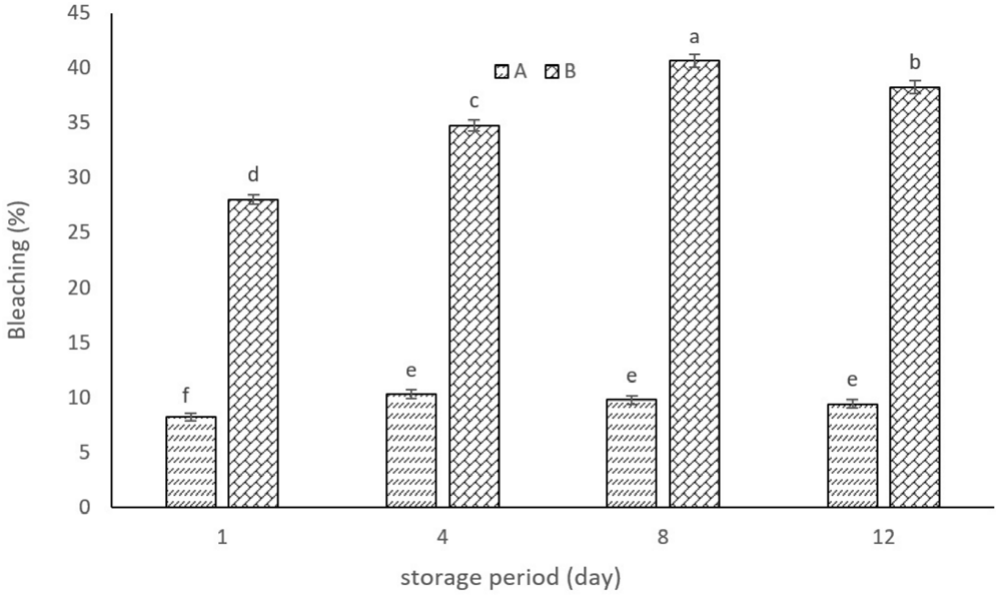
Bleaching of β‐carotene: Linoleic acid percentage in YCPAE (B) and YCPAE (A) during the storage period (12 days). YCPAE (yogurt containing pomegranate peel anthocyanin extract) and YWPAE (yogurt without pomegranate peel anthocyanin extract). Different letters indicate significant differences (*p* < 0.05, *n* = 3).

YCPAE exhibited a high antioxidant property in this test, showing a significant difference from YWPAE, indicating the superior antioxidant property of YCPAE. Also, the results show that the antioxidant property increased during storage until the 8th day but decreased on the 12th day (Figure 4). Researchers reported that yogurt containing (5%–20%) rose hips improved the antioxidant activity of yogurt (Sahingil and Hayaloglu 2022). Researchers reported that increasing the concentration of pomegranate peel extract increased the antioxidant activity of curd and also increased the shelf life of curd for 1 week (5°C) (Sandhya et al. 2018). However, Kharchoufi et al. (2017) reported a decrease in the antioxidant activity of Greek yogurt containing pomegranate extract during storage. Oxygen contact with anthocyanins and the formation of polyphenol‐protein complexes lead to a decrease in their antioxidant activity. However, polyphenol‐protein complexes can be reversible depending on factors such as product pH, ambient temperature, and protein and bioactive compound concentrations.

#### 3.3.8 FRAP Assay

**FIGURE 5:**
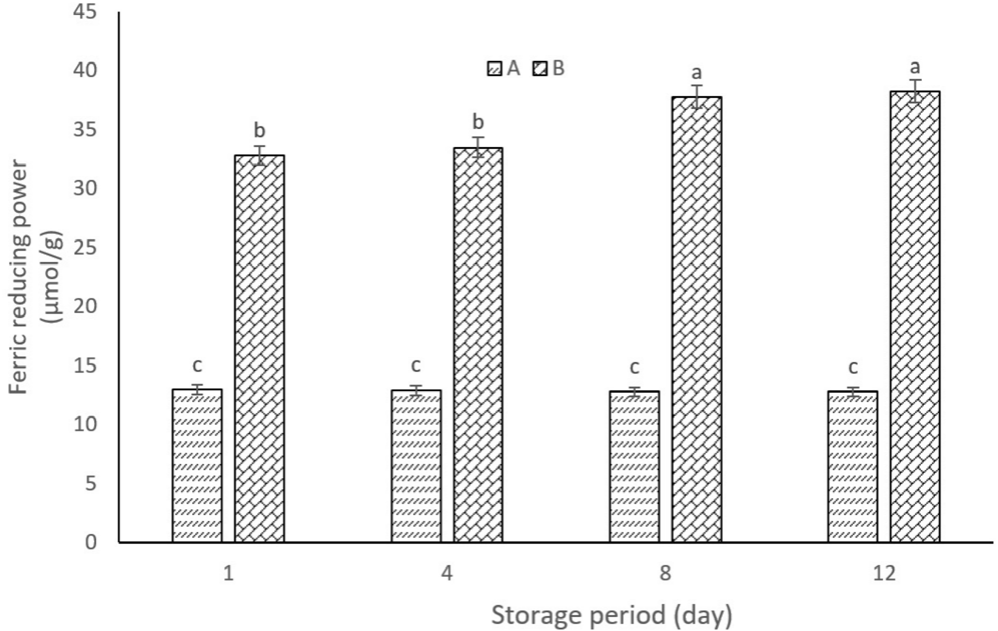
Ferric reducing power percentage in YCPAE (B) and YCPAE (A) during the storage period (12 days). YCPAE (yogurt containing pomegranate peel anthocyanin extract), and YWPAE (yogurt without pomegranate peel anthocyanin extract). Different letters indicate significant differences (*p* < 0.05, *n* = 3).

YCPAE showed high ferric reducing antioxidant power properties and was significantly different from YWPAE (Figure 5). Also, the antioxidant property of YCPAE increased during storage. However, there were no significant differences on days 1 and 4, as well as on days 8 and 12. Anthocyanins become more active and stable over time with increased acidity, decreased pH, and enhanced antioxidant properties. Szołtysik et al. (2021) analyzed yogurt enriched with grape polyphenol extract, where the polyphenol compounds in yogurt led to an increase in antioxidant potential and caused the purple color of yogurt to be pleasant. Also, although anthocyanins decreased during 2 weeks of yogurt storage, their antioxidant power to reduce Ferric was stable. Recent studies have shown that yogurt drinks enriched with berry extract and black grape pomace extract had higher antioxidant activity and higher amounts of polyphenolic compounds and anthocyanins than control yogurt. Also, storage time affected the stability of the antioxidant potentials of anthocyanins in yogurt with berry extract (Raikos et al. 2018). Thoughtfully incorporating natural plant extracts into food products not only amplifies their antioxidant properties but also contributes to extending their shelf life, ultimately creating a nourishing and health‐conscious choice that benefits overall well‐being. Adding plant extracts to food products increases their antioxidant properties and increases their shelf life (Dehghan et al. 2020).

#### 3.3.9 Microbial Analysis

The antimicrobial activity of YCPAE was investigated on mold, yeast, *P. aeruginosa* , and *S. aureus* . According to Table 3, the obtained YCPAE has a very high antimicrobial activity. In YCPAE, unlike YWPAE, mold and yeast were not observed during the storage days (12 days). The results also showed that YWPAE contained *P. aeruginosa* on days 1, 4, 8, and 12. However, *S. aureus* was observed on days 8 and 12. YCPAE contained *P. aeruginosa* on days 8 and 12. However, *S. aureus* bacteria were observed only on the 12th day, and the extract in yogurt prevented *P. aeruginosa* bacteria from being observed on the first 4 days and *S. aureus* bacteria on the first 8 days. Storing yogurt at refrigerator temperature caused more activity of *P. aeruginosa* because the bacterium is psychrophile. However, *S. aureus* bacteria are sensitive to cold, so they were less active. As a result, the effect of YCPAE on *S. aureus* bacteria is higher. Using bioactive waste as a preservative has better performance and fewer side effects than chemical food preservatives (Azmat et al. 2024). Research has shown that gram‐positive bacteria are more sensitive to these compounds than gram‐negative bacteria. Pomegranate peel extract in yogurt smoothies resulted in the elimination of *Candida*, *Aspergillus*, and *Penicillium* strains due to its antifungal activity (Alsubhi et al. 2022). *P. aeruginosa* , having a peptidoglycan wall and an outer membrane, is more resistant to the antimicrobial activity of the extract than *S. aureus* (Duffy and Harvilicz 2001; Alsubhi et al. 2022). In a study, cheese containing pomegranate peel extract had a lower number of *S. aureus* bacteria than control cheese during storage for 2 weeks (Parafati et al. 2021). Similarly, our study confirmed the antimicrobial effect of pomegranate peel extract. Ahmed and Abd Elhafez (2025) observed the elimination of coliforms in yogurt enriched with pomegranate peel extract (concentration 1%–1.5%). In a research, Peñafiel et al. (2018) investigated the use of two methods of extracting natural color (anthocyanin) from blackberry and its use in yogurt. The results showed that microbiological analyses, such as coliform, fungus, and yeast, were also in permissible amounts. Studies have documented an increase in the shelf life of a curd containing PoPAE for 1 week compared to control curd stored at refrigerated temperature (Sandhya et al. 2018). Kowaleski et al. (2020) studied the formulation of yogurt with strawberries. The results showed that the yogurt containing strawberry extract was free of mold and yeast, indicating that the antimicrobial properties of the anthocyanin extract were similar to the performance of PoPAE. PoPAE in a yogurt *smoothie* resulted in the elimination of Candida, Aspergillus, and Penicillium strains due to its antifungal activity (Alsubhi et al. 2022).

**TABLE 3:** Antimicrobial activity of YCPAE and YWPAE on mold, yeast, *Pseudomonas aeruginosa* , and *Staphylococcus aureus* during storage period (12 days).

Day	Yeast		Mold		*P. aeruginosa*		*S. aureus*	
	YWPAE	YCPAE	YWPAE	YCPAE	YWPAE	YCPAE	YWPAE	YCPAE
1	0.0 ± 0.00^a^	0.0 ± 0.00^a^	0.0 ± 0.00^a^	0.0 ± 0.00^a^	0.66 ± 0.47^a^	0.0 ± 0.00^b^	0.0 ± 0.00^a^	0.0 ± 0.00^a^
4	1.0 ± 0.00^a^	0.0 ± 0.00^b^	0.33 ± 0.47^a^	0.0 ± 0.00^b^	1.0 ± 0.81^a^	0.0 ± 0.00^b^	0.0 ± 0.00^a^	0.0 ± 0.00^a^
8	1.33 ± 0.47^a^	0.0 ± 0.00^b^	1.0 ± 0.81^a^	0.0 ± 0.00^b^	1.66 ± 0.94^a^	0.33 ± 0.47^a^	2.0 ± 0.81^a^	0.0 ± 0.00^b^
12	2.66 ± 0.47^a^	0.0 ± 0.00^b^	1.33 ± 0.94^a^	0.0 ± 0.00^b^	1.66 ± 1.24^a^	0.66 ± 0.47^a^	2.33 ± 1.24^a^	0.66 ± 0.47^b^

*Note:* Different letters in the row indicate significant differences (*p* < 0.05, *n* = 3).

Abbreviations: YCPAE, yogurt containing pomegranate peel anthocyanin extract; YWPAE, yogurt without pomegranate peel anthocyanin extract.

#### 3.3.10 Sensory Evaluation

**FIGURE 6:**
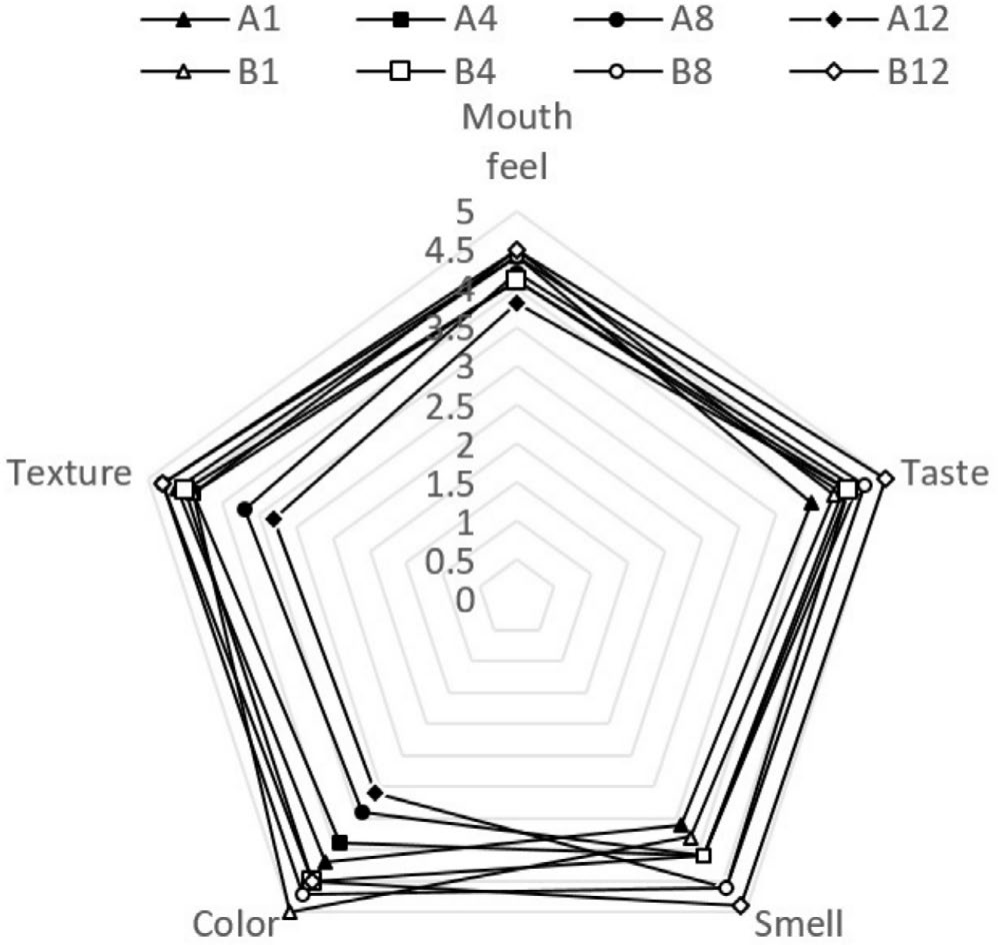
Sensory evaluation of YCPAE (A) and YWPAE (B) during the storage period (12 days). YCPAE (A1), yogurt containing pomegranate peel anthocyanin extract on day 1; YCPAE (A4), yogurt containing pomegranate peel anthocyanin extract on day 4; YCPAE (A8), yogurt containing pomegranate peel anthocyanin extract on day 8; YCPAE (A12), yogurt containing pomegranate peel anthocyanin extract on day 12; YWPAE (B1), yogurt without pomegranate peel anthocyanin extract on day 1; YWPAE (B4), yogurt without pomegranate peel anthocyanin extract on day 4; YWPAE (B8), yogurt without pomegranate peel anthocyanin extract on day 8; YWPAE (B12), yogurt without pomegranate peel anthocyanin extract on day 12; (*p* < 0.05). *n* = 10.

The results (Figure 6) showed that the smell and taste in YCPAE got a higher score over time. But the color, texture, and mouthfeel decreased over time. The YWPAE scored higher than the YCPAE. The texture, smell, and taste of YWPAE also gained more points with time. Over 12 days, the scores for odor and taste increased, whereas the scores for color, texture, and mouthfeel decreased. Researchers mixed different PoPAE at percentages (10.5%, 15%, 20%, 25%, 30%, and 35%) into milk before inoculating it with a yogurt starter culture. They reported that the mixing extract had good sensory properties, similar to the control yogurt (El‐Said et al. 2014). This highlights the remarkable potential of PoPAE not only to enhance the flavor profile and visual appeal of yogurt but also to elevate its nutritional value without compromising quality. In research on yogurt containing jabuticaba peel powder extract, it was stated that the presence of the extract reduced the a* parameter of yogurt (Machado et al. 2023). Sensory evaluation on the last day of storage of yogurt containing pomegranate juice showed that it was better in terms of taste, texture, and overall acceptance than the control yogurt (Ahmed and Abd Elhafez 2025). Sensory evaluation of low‐fat yogurt with 1% pomegranate peel obtained the lowest scores in taste, texture, appearance, and overall acceptance (Ibrahim et al. 2020).

## 4 Conclusion

Plant waste (PoPAE) with antimicrobial and antioxidant properties is a good alternative to synthetic additives (coloring, antioxidant, flavoring, preservative) in widely used fermented dairy products such as yogurt. As a result, an anthocyanin extract was prepared from pomegranate peel using microwave‐assisted extraction and used in a set of yogurt formulations. Its physicochemical, antioxidant, and antimicrobial properties were compared with YWPAE. 600 ppm was the lowest concentration of PoPAE and had more excellent antioxidant activity than 100 ppm TBHQ. Therefore, its concentration of 600 ppm was used in the yogurt formulation. Anthocyanin extract increased pH, syneresis and decreased acidity, viscosity, and lightness (L) in set yogurt. YCPAE demonstrated higher antioxidant power (DPPH, FRAP, βC/LA bleaching) compared to YWPAE. YCPAE prevented the presence of mold and yeast in yogurt during storage days (12 days). It also had a positive effect on reducing the activity of *P. aeruginosa* bacteria and *S. aureus* bacteria. At the beginning of the storage days, YCPAE had a lower sensory characteristic than YWPAE. However, with the increase in storage days, its sensory characteristics improved. The current study demonstrated that natural pigments (PoPAE) are a natural compound with useful applications in dairy products, including yogurt. Because it increased the antioxidant and antimicrobial properties, improved the physicochemical properties, increased shelf life, and increased the nutritional value of set yogurt.

## Author Contributions

**Niloofar Zahed:** formal analysis (equal), methodology (equal), software (equal), visualization (equal), writing – original draft (equal). **Reza Esmaeilzadeh Kenari:** conceptualization (equal), data curation (equal), investigation (equal), project administration (equal), supervision (equal), writing – review and editing (equal).

## Conflicts of Interest

The authors declare no conflicts of interest.

## Data Availability

The data from this research are confidential and will not be shared.
